# Reply to S. Rastogi et al

**DOI:** 10.1200/JGO.2016.006775

**Published:** 2016-10-12

**Authors:** Seema Gulia, Sudeep Gupta

**Affiliations:** **Seema Gulia** and **Sudeep Gupta**, Tata Memorial Centre, Mumbai, India

We thank Rastogi and Aggarwal^1^ for their interest in our recent article that proposes a framework for organization of chemotherapy and systemic therapy services in India.^2^ They have highlighted some relevant practical difficulties in integrating oncology services into the health care infrastructure of India. However, we disagree with their contention that oncology care cannot be integrated within existing public sector health care facilities. Several indications in India’s health indices, affected by the same health care system, have shown steady improvement. A consistent decline in the infant mortality rate (IMR) has been seen in India, from 165 per 1,000 live births in 1960 to 38 per 1,000 in 2015.^3^ The maternal mortality rate (MMR) has been reduced from 556 per 100,000 live births in 1990 to 174 per 100,000 in 2015.^4^ The reduction in IMR and MMR strongly reflects the overall effectiveness of the health care system in India. Rastogi and Aggarwal contend that the rural health program in India has miserably failed, which is not true. Since the launch of the National Rural Health Mission in 2005, > 157,000 personnel have been used in the health sector, the Janani Suraksha Yojana has been successful in ensuring peripartum care of > 150 million women in government facilities, and > 600,000 newborns receive care in neonatal units in district hospitals every year.^5^ Furthermore, by using the same health care infrastructure, Kerala (one of the Indian states) attained an IMR and MMR (12 in 1,000 and 66 in 100,000, respectively) comparable with that of developed countries.^6^ Recently, the health sector in India has seen considerable improvement in human resource availability. Various strategies, such as compulsory rural service, linkage of rural service to postgraduate education, and provision of monetary incentives, have been instituted to increase the availability of physicians in underserved rural areas. A substantial increase in number of undergraduate and postgraduate seats in medical colleges across India has occurred. Furthermore, financial support is provided to states under the National Rural Health Mission to strengthen the health system, including engagement of nurses, physicians, and specialists.^7^

The most effective health care models around the world have not created separate verticals for every disease, and this is unlikely to be a viable strategy in the long run. In keeping with this experience from other countries, we have proposed maximal use of the existing public sector health care delivery system in India to undertake safe and effective delivery of chemotherapy to patients with cancer. Recently, our institution has partnered with the Government of Maharashtra to initiate the delivery of chemotherapy for breast, cervical, and oral cancers at five district hospitals, which will be extended to 24 districts in the next 3 years.^8^ Of note, a gap analysis of infrastructure and human resources within this venture has used elements of our proposal. We do not believe, unlike Rastogi and Aggarwal, that the need for premedication, hydration, and potential problems like extravasation are insurmountable challenges in initiating chemotherapy services in district hospitals, and our confidence is shared by many partners in public and private health care domains.

Perhaps the biggest challenge in India is the disparity in human development indices and level of infrastructure among various regions and states. Therefore, we have proposed human resource and infrastructure recommendations at each health care level to guide the allocation of resources for safe delivery of chemotherapy in various scenarios.^2^ In this context, we agree with Rastogi and Aggarwal that the innovative use of modern information technology and efficient record keeping are important to the integration of oncology health care delivery at various levels.

Although several constraints exist, with hope, optimism, and careful, cost-effective, hierarchical planning, India will improve its health care (including oncology) delivery mechanisms. An overly pessimistic attitude is unlikely to facilitate meaningful change in the long run.
